# Estimating the Number of Antiretroviral Treatment Facilities Based on the Wilson–Blower Method

**DOI:** 10.1371/journal.pmed.0020270

**Published:** 2005-08-30

**Authors:** Ntambwe Malangu

**Affiliations:** **1**University of LimpopoPretoriaSouth Africa

The implementation of the comprehensive plan for the care, management, and treatment of HIV and AIDS in South Africa [1] needs to be supported by all. It is encouraging to note that Wilson and Blower [2] used South Africa to develop a novel method to determine the optimal strategy for allocating antiretroviral treatment (ART) sites among health-care facilities (HCFs) in KwaZulu–Natal.

An equitable allocation of HCFs is necessary to ensure that each individual with HIV will have an equal chance of receiving antiretroviral drugs (ARVs). We have applied their method to determine the number of ART HCFs per district in KwaZulu–Natal.

We first set out to assemble basic details about the KwaZulu–Natal health districts, namely, population and number of hospitals and fixed and mobile clinics. Secondly, by using population data as reported in the KwaZulu–Natal Department of Health 2004 annual report [3], and based on a 10% HIV prevalence, we determined the HIV population per district and also as a percentage of the total HIV population in the province. Finally, we calculated the number of ART HCFs, based on the premise that 54 ART HCFs will serve 100% of the HIV population in the province. By contrasting the estimated number of ART HCFs with the current ART HCFs, we calculated the number of ART HCFs that still need to be established.

The national target for access to HCFs is 10,000 habitants per one fixed primary health-care (PHC) facility [2]. A PHC facility could be a clinic, a community health center, or a hospital. At present, there is one fixed PHC facility for 17,215 inhabitants in KwaZulu–Natal [3].

With regard to ART, the 54 HCFs proposed by Wilson and Blower translate to 18,076 people with HIV per facility (Table 1). This number is close to the actual figure of 17,215 inhabitants per facility in the province.

**Table 1:**
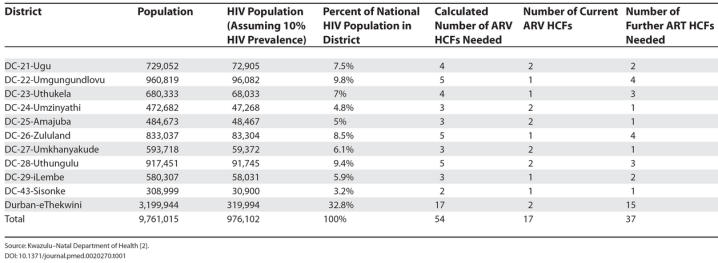
Number of ART HCF Calculated for Each District

Source: Kwazulu–Natal Department of Health [2].

In terms of equity, it could be argued, for instance, that the two facilities in the eThekwini district cannot be expected to provide ARVs to the estimated 319,994 individuals with HIV. In comparison, the Umziyathi, Amajuba, Umkhanyakude, Uthungulu, and Ugu districts currently have the same number of ART HCFs as eThekwini, but serve smaller populations (Table 1). This reflects the fact that the choice of the current facilities was guided more by practical considerations, such as availability of staff and infrastructure, than by the principle of equity as suggested by the World Health Organization [4].

From our calculations, it seems that in order to achieve treatment equity for individuals with HIV in KwaZulu–Natal, more ARV HCFs should be established as follows: 15 in eThekwini, four each in Umgungundlovu and Zululand, three each in Uthukela and Uthungulu, two each in Ugu and iLembe, and one each in Amajuba, Sisonke, Umzinyathi, and Umkhanyakude.

As a recommendation, future rollout of ART should take into consideration the principle of equity. This will ensure that all people with HIV have equal access to ARVs from their nearest HCF. We show that by applying the Wilson–Blower method, it is possible to determine the number of health-care facilities where ARVs would be equitably provided.

*This correspondence letter was peer reviewed.*
